# Low *TNFAIP3* expression in psoriatic skin promotes disease susceptibility and severity

**DOI:** 10.1371/journal.pone.0217352

**Published:** 2019-05-23

**Authors:** Nahla Yassin Sahlol, Marwa Salah Mostafa, Lamiaa Abd El-Fattah Madkour, Dina Metwally Salama

**Affiliations:** 1 Department of Microbiology and Immunology, Faculty of Medicine, Cairo University, Cairo, Egypt; 2 Department of Dermatology, Faculty of Medicine, Cairo University, Cairo, Egypt; Celcuity, UNITED STATES

## Abstract

Psoriasis vulgaris is a systemic disorder with an underlying immune dysregulation that predisposes to inflammatory skin lesions. Meanwhile, tumor necrosis factor alpha-induced protein 3 (TNFAIP3) has been described as a protective molecule against the deleterious effects of uncontrolled inflammation. In this study, we compared the expression levels of *TNFAIP3* in blood and psoriatic skin biopsies from psoriatic patients *versus* those in normal individuals. Additionally, the levels of TNFAIP3 protein in psoriatic skin biopsies were compared to those in normal individuals. Thirty psoriatic patients and 30 healthy participants (control group) were enrolled. The expression levels of *TNFAIP3* in blood and skin were measured by quantitative reverse transcription PCR, while the skin levels of TNFAIP3 protein were measured by western blot. Psoriatic patients showed significantly lower expression levels of *TNFAIP3* in psoriatic skin and blood (*P*< 0.001) as well as of TNFAIP3 protein in psoriatic skin (*P*< 0.001) compared to controls. A significant lower expression of *TNFAIP3* and TNFAIP3 protein in psoriatic skin was detected in moderate/severe cases compared to mild cases (*P* = 0.004 and 0.003 respectively). Moreover, a significant negative correlation was found between *TNFAIP3* mRNA in psoriatic tissue and psoriasis area severity index values (r_s =_ -0.382, *P-*value = 0.037).

In conclusion, TNFAIP3 may serve as a predictive and prognostic biomarker in psoriatic patients. Enhancing the expression and/or function of *TNFAIP3* in the affected cell type may be a promising therapeutic strategy.

## Introduction

Psoriasis vulgaris is an autoimmune papulosquamous disorder [1] characterized by inflammatory skin manifestations and sustained activation of multiple immune cells [2]. It is a spectrum of disease with physical, psychological, and social impacts on patients [3]. About 30% of patients develop an inflammatory arthritis [4], resulting in physical disability [3]. Occasionally, psoriatic patients may develop uveitis, inflammatory bowel disease [4], chronic pulmonary disease, liver and renal disease [5, 6], stroke and myocardial infarction [7]. It is also a psychiatric stressor; where psoriatic patients have a higher prevalence of alcoholism and depression [8].

In psoriatic patients, keratinocytes are the key participants in innate immunity recruiting T cells to the skin, and these T cells play a crucial role in sustaining disease activity. Activated T cells produce abundant psoriatic cytokines including interleukin-17 (IL-17), IL-22, interferon-γ (IFN-γ) and tumor necrosis factor (TNF)-α [9]. These cytokines mediate effects on keratinocytes to amplify psoriatic inflammation [10] which is paralleled with disease severity [11].

In the same context, genome-wide association studies (GWAS) using DNA microarray chips showed that the genome regions mostly related to psoriasis development are associated with the immune system, including *TNF-*𝛼, *IL-12B*, *IL-23R* and the human leukocyte antigen Cw6 (*HLA-Cw6*) of the major histocompatibility complex (*MHC*) [12].

Meanwhile, accumulating evidence suggests that abnormal immune regulatory genes are involved in the molecular pathogenesis of psoriasis vulgaris [13]. One of those genes that has been described as a potent contributor to psoriasis pathogenesis is the tumor necrosis factor alpha-induced protein 3 (*TNFAIP3*) gene [14], which encodes TNFAIP3 protein, also known as TNF-α- inducible zinc finger protein A20 [15].

Nonetheless, *TNFAIP3* overexpression has no effect on keratinocyte differentiation, implying that TNFAIP3 is not a direct modulator of this differentiation. On the other hand, *TNFAIP3* overexpression in keratinocytes significantly represses cytokine production, suggesting a potential role of TNFAIP3 deficiency in the development of psoriasis *via* sensitization of keratinocytes to external stimuli [16].

The expression of *TNFAIP3*, which takes place in both the cytoplasm and the nucleus of all cell types, is induced by TNF-α through nuclear factor kappa-light-chain-enhancer of activated B cells (NF-κB)-dependent signals [17,18]. Under normal conditions, *TNFAIP3* is expressed in low quantities, whereas in the event of inflammation, its expression is spectacularly prompted by TNF-α and NF-κB activation [19]. Therefore, in immune cells, TNFAIP3 can be induced strongly under inflammatory conditions and acts as a negative-feedback regulator of NF-κB activation [20, 21]. Meanwhile, a progressively high *TNFAIP3* expression inhibits TNF-induced NF-κB signaling in a dose-dependent fashion [22–24].

Given the facts that (1) the differentiation of keratinocytes is dependent on NF-κB signaling [25], (2) increased expression of NF-κB-dependent gene products is associated with skin inflammation and psoriasis [15, 26] and (3) the pleiotropic functions of NF-κB and cell-death signaling in various cell types that contribute to autoimmune and inflammatory diseases, it can be hypothesized that the dysregulation of NF-κB inhibitory signaling cascades may contribute to disease pathogenesis [16]. Hence, the hypomorphic expression or function of *TNFAIP3* may increment a susceptibility to psoriasis [27]. It is worth mentioning that selective deletion of *TNFAIP3* in mice has been linked to the development of inflammatory pathologies, keratinocyte hyperproliferation, disheveled hair and sebocyte hyperplasia [28].

It has also been noticed that *TNFAIP3* gene single nucleotide polymorphisms (SNPs) are strongly associated with psoriasis through their inhibitory effect on cellular *TNFAIP3* expression [29, 30]. A meta-analysis of the *TNFAIP3* region has recognized 49 variants; the most important being rs582757 followed by rs6918329. Analysis of *TNFAIP3* haplotypes revealed that the psoriasis risk haplotype is different from other autoimmune diseases [31]. Meanwhile, the protective anti-inflammatory role of TNFAIP3 against psoriasis can be plausibly explained by its ability to suppress inflammasome activity and subsequently cell death [32–34].

The current therapeutic lines for psoriasis include systemic and biologic agents; however, most of them are of questionable efficacy and/or high toxicity [35]. Furthermore, the financial cost of treatment with the anti-TNF drugs is considerably higher than traditional systemic therapies for psoriasis such as phototherapy and methotrexate [36]. A recent pharmacogenetic study reported that homozygous patients for an SNP rs610604 in *TNFAIP3* showed an inferior response to ustekinumab, a biologic used in psoriasis, in comparison with a control group [37].

Therefore, studies to identify accurate predictors of therapeutic response in psoriasis would be of great value in making decisions on treatment options [15]. Development of strategies to induce *TNFAIP3* expression is required to provide effective therapy. However, therapeutic approaches usually use drugs that interfere with the functions of target proteins rather than activating them. Hence, it is proposed to target molecules that suppress *TNFAIP3* expression and/or function. Studies have identified microRNAs that target *TNFAIP3* gene; thus, inhibition of these microRNAs could enhance *TNFAIP3* expression [38–40].

TNFAIP3 may serve as a potential molecular marker to anticipate the response of treatment with TNF-α–inhibitors [15] as well as a potential new line of treatment. Previous studies were limited by the sole use of quantitative reverse transcription polymerase chain reaction (QRT-PCR) for measurement of *TNFAIP3* gene expression, as well as by lack of data regarding TNFAIP3 protein and data from local psoriatic tissues [41]. Consequently, the quantification of TNFAIP3 protein was an additional aim in our research work.

The aim of this study was to compare the expression levels of *TNFAIP3* gene in blood samples and psoriatic skin biopsies from patients with those in normal individuals. It also aimed at comparing TNFAIP3 protein levels in psoriatic skin biopsies from patients with that in normal individuals. The effect of the expression of *TNFAIP3* in blood samples and psoriatic skin biopsies from psoriatic patients on the severity of psoriasis was also studied.

## Material and methods

This case control study was conducted on thirty psoriatic patients attending the psoriasis outpatient clinic at the Dermatology Department of Kasr Al-Ainy Hospitals, Cairo University, during the period from February 2017 to December 2017. Patients with erythrodermic or pustular psoriasis, pregnant and lactating females, patients with autoimmune diseases e.g. systemic lupus erythematosus, or major systemic diseases, and patients receiving any immunosuppressive drug were excluded from the study. Thirty healthy participants matched for age and gender were enrolled as a control group. Before commencement of the study, it was approved by the Research Ethics Committee of the Institutional Review Board, Faculty of Medicine, Cairo University. An informed consent was obtained from each participant.

Detailed history was taken from each patient including personal history, history of the present illness, precipitating factors and intake of any medications. Clinical assessment of patients to determine the disease severity using psoriasis area severity index (PASI) score was performed by the same dermatologist.

### 1. Specimen collection and transport

Blood samples obtained from each participant were collected in EDTA tubes for measuring the expression levels of the *TNFAIP3* gene by QRT-PCR. Four mm full thickness skin biopsy was obtained from the psoriatic tissue in each patient for quantitative detection of the expression levels of each of *TNFAIP3* gene by QRT- PCR and TNFAIP3 protein by western blot technique. Blood samples and biopsies of normal skin were collected from 30 healthy participants admitted to the Plastic Surgery Department, Kasr Al-Ainy Hospital for plastic surgical procedures involving removal of a skin portion. All recruits had no history of chronic dermatological or systemic disease including renal diseases, liver diseases, malnutrition or autoimmune diseases. They were informed about the study aim and protocol. Skin biopsies were collected in sterile eppendorf tubes. All samples were kept frozen at -80°C on lysis solution (Thermo Fisher Scientific, USA) to immediately preserve the gene expression profile until further testing by QRT-PCR which was performed at the Unit of Molecular Biology of the Medical Biochemistry Department, Faculty of Medicine, Cairo University.

### 2. Quantitative reverse transcription PCR

Total RNA was extracted from blood using Qiagen RNA blood extraction kit (Qiagen, USA) according to the manufacturer's instructions. Total RNA was also extracted from psoriatic tissue homogenate using Qiagen tissue extraction kit (Qiagen, USA) according to the manufacturer's instructions. The total RNA (0.5–2 μg) was utilized for cDNA conversion employing high capacity cDNA reverse transcription kit (Fermentas, USA). The expression levels of TNFAIP3 were assessed using the primer sets shown in Table 1.

**Table 1:** Primer sequences of the studied genes.

Gene	Primer sequence
***TNFAIP3***	Forward primer: 5ʹ-CG TCCAGGTTCCAGAACACCATTC-3ʹ Reverse primer: 5ʹ-TGCGCTG GCTCGATCTCAGTTG-3ʹ
***β-actin***	Forward primer: 5’-GACTACCTCATGAAGATCCTCACC-3ʹ Reverse primer:5ʹ-TCTCCTTAATGTCACGCACGATT-3ʹ

The primers were purchased from *Bio Basic* Inc., Canada and SYBR Green I kit (Qiagen, USA) was employed according to the manufacturer's instructions. For each sample, a total of 25 μl reaction volume was prepared by adding the following reagents: 12.5 μlof Syber green mix, 1 μl of each primer, 5 μl of the cDNA template, and 5.5 μl RNAse free water. The prepared reaction mixtures were processed in the Applied Biosystems *StepOne* Thermal Cycler with software version 3.1 (Applied Biosystems, USA) for amplification and analysis. The cycling conditions were as follows: hold phase at 50°C for 2 min followed by 40 cycles of denaturation at 95°C for 15 sec, annealing at 60°C for 1 min and extension at 72°C for 1 min.

The relative quantity (RQ) of *TNFAIP3* expression level was assessed relative to the housekeeping gene (*β-actin*), which was used as an internal control and was expressed as 2^-ΔΔCt^.

### 3. Estimation of TNFAIP3 protein by western blot technique

Skin biopsies were lysed in Radioimmune precipitation assay (RIPA) lysis buffer PL005 (Bio BASIC, Canada). Total protein was measured using Bradford Protein Assay Kit for quantitative protein analysis (BIO BASIC, Canada) according to the manufacturer's instructions. Samples were run on sodium dodecyl sulfate (SDS)-polyacrylamide gel using TGX Stain-Free FastCast Acrylamide Kit (Bio-Rad, USA), then transferred onto nitrocellulose membranes and incubated with TNFAIP3 antibodies (Thermo Fisher Scientific, USA). Blots were then incubated with peroxidase-conjugated secondary antibodies (Novus Biologicals, Canada).The chemiluminescent substrate (Clarity Western ECL substrate—BIO-RAD, USA) was applied to the blot according to the manufacturer’s recommendations. The chemiluminescent signals were captured using a charge-coupled device (CCD) camera-based imager (BIO-RAD, USA). Image analysis software was used to read the band intensity of the target proteins relative to a control sample by total protein normalization on the ChemiDoc MP imager.

### Statistical analysis

Data were analyzed using SPSS version 25. Data were summarized using mean, standard deviation, minimum and maximum in quantitative data, and using frequency (count) and relative frequency (percentage) for categorical data. Comparisons between the expression levels of *TNFAIP3* gene and the TNFAIP3 protein in cases and controls as well as within patient groups were done using the non-parametric Mann-Whitney test. For comparing PASI score, Chi square (χ2) test was performed. Correlations between expression levels of *TNFAIP3* gene as well as the TNFAIP3 protein in the cases and controls and within patient groups were done using Spearman correlation coefficient (r_s_). *P*-values ≤ 0.05 were considered statistically significant.

## Results

### 1. Demographic and clinical data of psoriatic patients and control subjects

The present study was conducted on 30 psoriatic patients, aged between 18–75 (41±17.72) and 30 healthy subjects, aged between 19–65 (44.30 ±12.83). The patients included 13 males (43.3%) and 17 females (56.7%), while the control group included 14 males (46.7%) and 16 females (53.3%).Clinical data of psoriatic patients are shown in Table 2.

**Table 2:** Clinical data of psoriatic patients.

	Cases: no (%)	
**Onset**	Sudden	1 (3.3%)
	Gradual	29 (96.7%)
**Course**	Remission and exacerbation	27 (90%)
	Progressive	3 (10%)
**Associated disease**	No	30 (100%)
**Family history**	No	30 (100%)
**Severity**	Mild	20 (66.7%)
	Moderate	5 (16.7%)
	Severe	5 (16.7%)
**Disease Duration (years)**	Range	0.5–20
	Mean ± SD	8.94 ± 6.67
**PASI score**	Range	0.7–28.50
	Mean ± SD	8.79 ± 8.33

PASI: Psoriasis area severity index

### 2. Comparison between TNFAIP3 in cases and controls

#### A. *TNFAIP3* gene expression levels in blood

The expression levels of *TNFAIP3* gene in blood were significantly lower in the psoriatic patients (mean ± SD = 0.39 ± 0.22) compared to the controls (mean ± SD = 1.04 ± 0.31) (*P*< 0.001) (Table 3).

**Table 3:** Comparison between TNFAIP3 levels in psoriatic patients and controls.

	Cases		Controls		*P-value*
	Mean ±SD	Range	Mean ±SD	Range	
***TNFAIP3 mRNA* in blood**	0.39 ± 0.22	0.06–1.01	1.04 ± 0.31	0.54–2.03	**< 0.001**
***TNFAIP3 mRNA* in skin**	0.30 ± 0.16	0.07–0.69	1.02 ± 0.21	0.69–1.48	**< 0.001**
**TNFAIP3 protein in skin**	0.42 ± 0.21	0.12–0.89	1.01 ± 0.02	0.99–1.05	**< 0.001**

TNFAIP3: Tumor necrosis factor alpha induced protein 3

#### B. *TNFAIP3 mRNA* expression levels in skin biopsies

The expression levels of *TNFAIP3* gene in skin were significantly lower in the psoriatic patients (mean ± SD = 0.30 ± 0.16) compared to the controls (mean ± SD = 1.02± 0.21) (*P*< 0.001) (Table 3).

#### C. TNFAIP3 protein levels in skin biopsies

**Fig 1:**
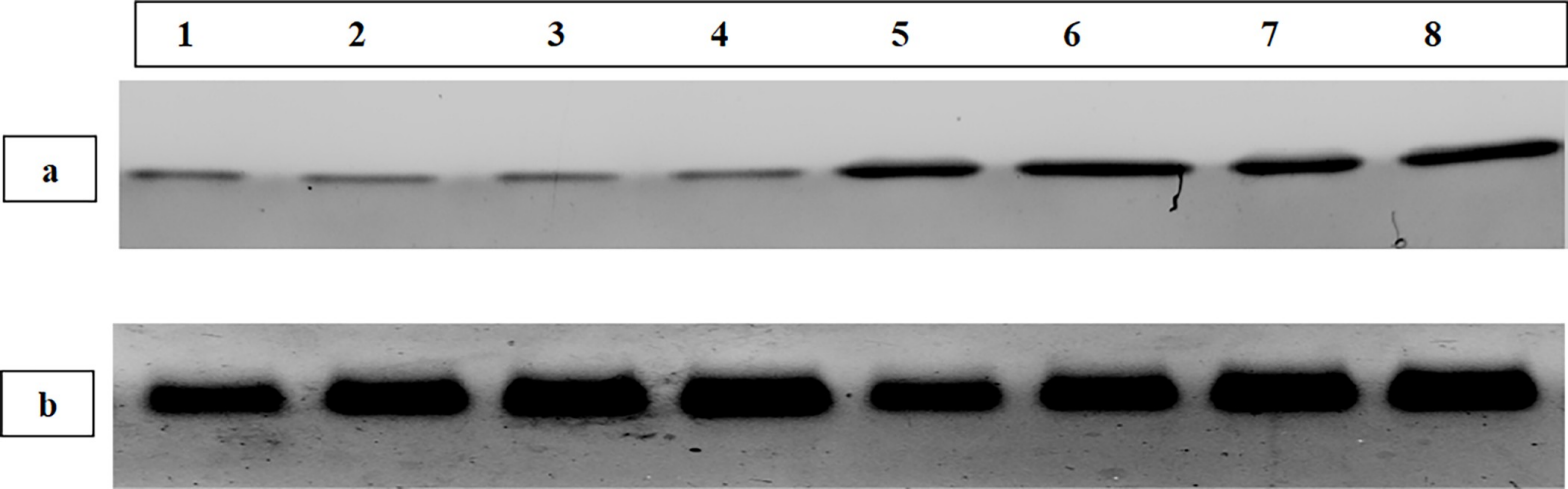
Western blot analysis showing: a) TNFAIP3 protein; and b) the housekeeping protein (β-actin) in skin biopsies. Bands 1–4 show TNFAIP3 (upper figure) and β-actin (lower figure) in the psoriatic skin lesions of patients number 2, 8, 12 and 15.Bands 5–8 show TNFAIP3 (upper figure) and β-actin (lower figure) in skin biopsies of the normal control subjects 1, 2, 3 and 4.

The expression levels of TNFAIP3 in skin were significantly lower in the psoriatic patients (mean ± SD = 0.42 ± 0.21) compared to the controls (mean ± SD = 1.01 ± 0.02) (*P* < 0.001) (Table 3 and Fig 1).

### 3. Relationship between TNFAIP3 and disease severity

**Fig 2:**
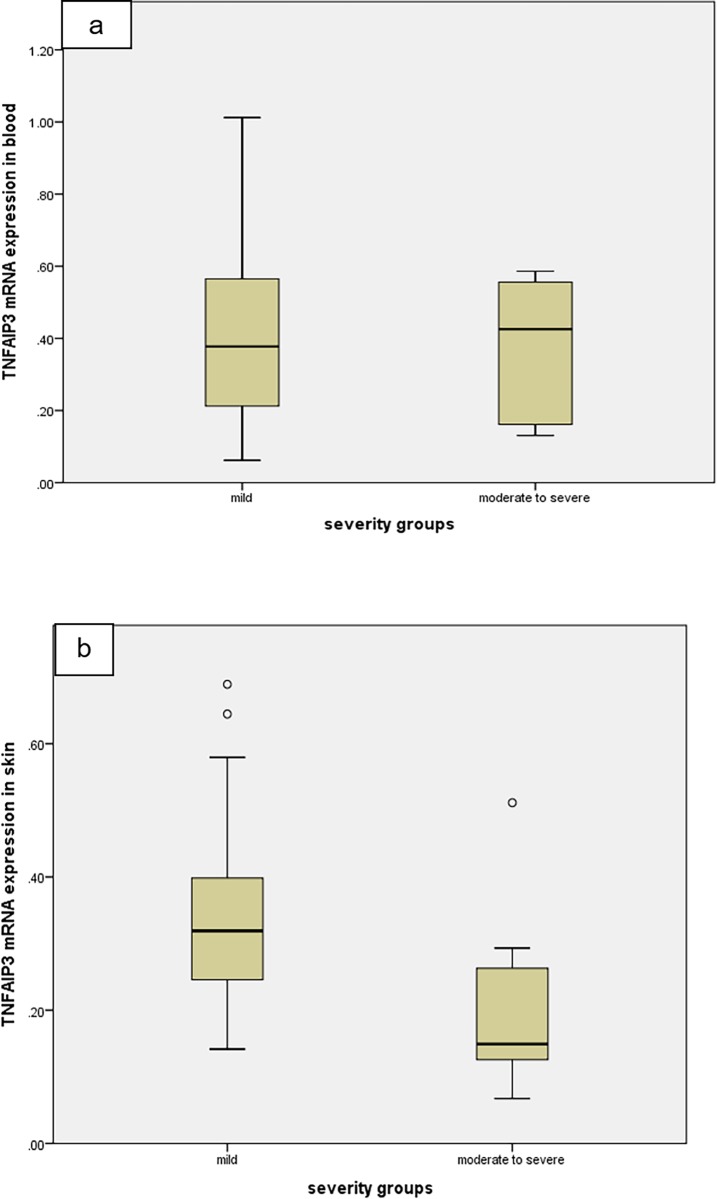
Comparison between mild and moderate/severe cases regarding *TNFAIP3* mRNA levels in blood and skin biopsies. (a) *TNFAIP3* mRNA in blood (*P* = 0.914) and (b) *TNFAIP3* mRNA in skin (*P* = 0.004). o = extreme cases.

**Fig 3:**
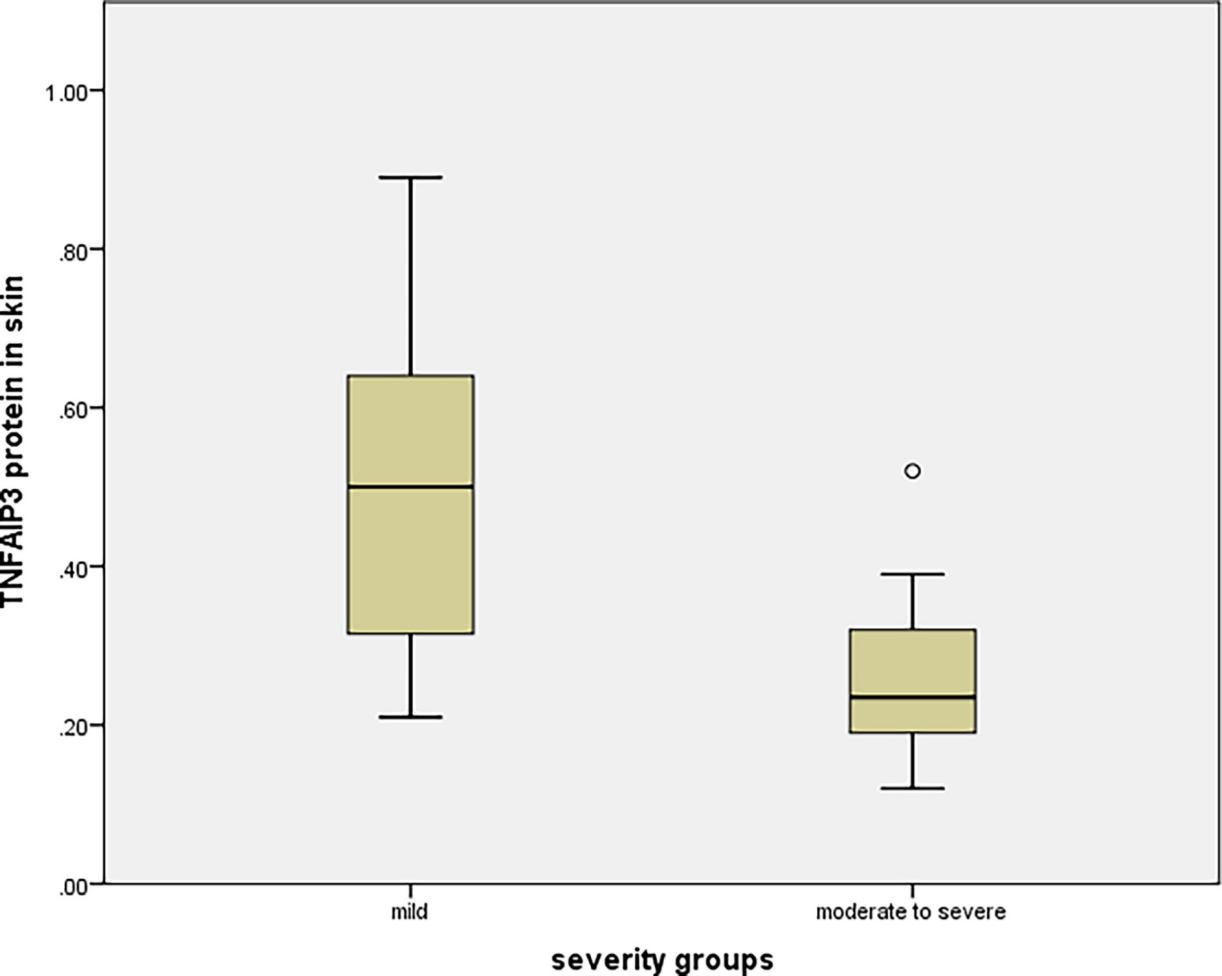
TNFAIP3 protein levels in psoriatic skin of mild and moderate/severe cases (*P* = 0.003). o = extreme case.

The 30 psoriatic patients involved in this study were classified according to disease severity into two groups: Group 1 containing mild cases with PASI < 10 (20 patients [66.7%]), and Group 2 containing moderate and severe cases with PASI ≥ 10 (10 patients [33.3%]). Statistically significant lower expression levels of *TNFAIP3* gene and TNFAIP3 protein in psoriatic skin lesions were detected in Group 2 compared to group 1 (*P* = 0.004 and *P* = 0.003, respectively). On the other hand, no statistically significant difference was detected in *TNFAIP3* gene expression in blood between the two groups (*P* = 0.914) (Table 4 and Figs 2 and 3).

**Table 4:** TNFAIP3 in mild and moderate/severe cases.

	Group 1* (n = 20)		Group 2** (n = 10)		*P- value*
	Mean ± SD	Range	Mean ± SD	Range	
***TNFAIP3 mRNA* in blood**	0.39 ± 0.25	0.06–1.01	0.39 ± 0.18	0.13–0.59	0.914
***TNFAIP3 mRNA* in skin**	0.35 ± 0.16	0.14–0.69	0.2 ± 0.13	0.07–0.51	**0.004**
**TNFAIP3 protein level in skin**	0.49 ± 0.21	0.21–0.89	0.27 ± 0.12	0.12–0.52	**0.003**

*Group 1: mild cases with PASI < 10

**Group 2: moderate/severe cases with PASI ≥ 10

TNFAIP3: Tumor necrosis factor alpha induced protein 3

Spearman correlation coefficient test showed that there was no correlation between disease severity indicated by PASI score with *TNFAIP3* gene expression in blood (r_s_ = -0.084, *P* = 0.657) or with skin TNFAIP3 protein (r_s_ = -0.292, *P* = 0.117) Meanwhile, a significant negative correlation was found between *TNFAIP3* mRNA in psoriatic tissue and disease severity (r_s =_ -0.382, *P* = 0.037).

### 4. Correlations between *TNFAIP3* in blood and psoriatic lesions

No correlations were detected between blood levels of *TNFAIP3* mRNA with each of skin levels of *TNFAIP3* mRNA (r_s_ = -0.224, *P* = 0.235) and skin levels of TNFAIP3 protein (r_s_ = -0.312, *P* = 0.093).

## Discussion

In order to develop efficient successful therapy for psoriasis vulgaris, a major advancement has been achieved in understanding the complex nature of the disease. Nevertheless, more studies are still warranted in this scope [42]. Hyper-activated NF-κB is one of the main factors causing inflammation during psoriasis. NF-κB is also considered the key regulator in the pathology of psoriasis, where multiple cells types, chemokines, and cytokines associated with psoriasis are dependent on the activation of NF-κB signaling [43]. This has led us to question the role of *TNFAIP3*, a feedback inhibitor of NF-κB activation, in psoriatic inflammatory processes.

In this study, we demonstrated that the expression of *TNFAIP3* gene was altered in psoriasis, as evidenced by a significant down-regulated expression of *TNFAIP3* mRNA in blood and skin of psoriatic patients compared to controls (*P <*0.001). We also found out that altered *TNFAIP3* mRNA expression was accompanied by a significant decrease in its protein expression in psoriatic skin biopsies compared to controls *(P <*0.001). These findings were in accordance with the findings of *Aki et al*. [44] who reported a reduced expression of *TNFAIP3* mRNA in skin biopsies from psoriatic patients in both involved and uninvolved skin. Based on the finding that low *TNFAIP3* expression is associated with increased susceptibility to inflammation, it can be concluded that decreased TNFAIP3 levels potentiates psoriasis susceptibility. Additionally, the fact that NF-κB stimulates *TNFAIP3* mRNA expression in response to inflammation confirms that low *TNFAIP3* expression acts as a driver rather than a result of the inflammatory process seen in psoriasis [44].

Meanwhile, TNFAIP3 protein has been found down-regulated in lesional epidermis of patients with psoriasis and atopic dermatitis, signifying that absence of *TNFAIP3* expression in keratinocytes triggers the generation of inflammatory skin conditions including psoriasis [16, 45]. Additionally, up-regulation of proinflammtory cytokines and chemokines, as well as systemic proinflammatory changes have been detected in epidermis-specific TNFAIP3 knockout mice, in which TNFAIP3 absence exacerbated the severity of experimental psoriasis. Hence, TNFAIP3 deficiency in keratinocytes leads to inflammatory gene signature in the epidermis and systemic proinflammatory changes and is sufficient to exacerbate inflammatory skin disorders [45].

Interestingly, *TNFAIP3* haplo-insufficient mice that were not exposed to inflammatory stimuli have not developed apparent dermatitis. Likewise, human studies indicate that low *TNFAIP3* expression reduces the threshold for dermatitis in psoriatic patients, whereas external factors such as infections and trauma are necessary to induce psoriatic lesions [44]. A reduced expression of *TNFAIP3* may sensitize to the development of inflammatory pathology possibly in collaboration with additional genetic defects or in specific environmental conditions [18]. *TNFAIP3* haplo-insufficiency has been also described as the cause of autoinflammatory manifestations by *Franco-Jarava et al*. [46] who identified a deletion of a 13 Mb on chromosome 6, including *TNFAIP3*, in a patient with psychomotor and growth delay, neutrophilic dermatosis, and recurrent orogenital ulcers. Moreover, heterozygous mutations in the *TNFAIP3* gene associated with *TNFAIP3* haplo-insufficiency have been identified in a Behcet–like illness [47]. Patients with *TNFAIP3* haplo-insufficiency have also shown elevated levels of proinflammatory cytokines, including TNF-α, IL-6, IL-18 and IFNγ–inducible protein-10 [48].

On the other hand, and on the contrary to our results, *Liu et al*. [49] showed that TNFAIP3 protein is over-expressed under psoriatic inflammation, favoring keratinocyte proliferation and suppressing apoptosis. This strong expression of *TNFAIP3* found in lesional psoriatic skin has been posited to inhibit the NF-κB pathway; however, a strong expression of NF-κB was observed [49], suggesting that *TNFAIP3* is disabled or dysfunctional in patients with psoriasis [50]. It is worth mentioning that in psoriatic patients with higher *TNFAIP3* expression levels in their skin lesions compared to normal controls, calcipotriol (a vitamin D3 analogue) therapy significantly reduced the expression levels of both *TNFAIP3* and NF-κB [49, 51]. A possible explanation is that in patients with dysfunctional TNFAIP3, an attempt of the body to over-express *TNFAIP3* in order to control inflammation results in TNFAIP3 elevated levels which are brought back to baseline levels in response to treatment.

In the present study, the levels of *TNFAIP3* mRNA and its protein in psoriatic skin lesions were significantly lower in group 2 (moderate/severe) compared to group 1 (mild) (*P-*value = 0.004 and 0.003 respectively). Moreover, a significant negative correlation was found between *TNFAIP3* mRNA in psoriatic tissue and PASI values (r_s =_ -0.382, *P-*value = 0.037). This inverse correlation between *TNFAIP3* gene and the severity of psoriasis suggests that increasing the expression and/or function of TNFAIP3 is a promising therapeutic strategy. Remarkably, a study by Ma and Malynn [52] showed that induction of *TNFAIP3* expression has provided protection against experimental colitis in mice. This finding confirms the anti-inflammatory effect of TNFAIP3.

On the other hand, no statistically significant difference was detected between the two groups regarding *TNFAIP3* gene expression in blood. No correlation was detected between blood levels of *TNFAIP3* mRNA and disease severity. This finding suggests that the *TNFAIP3* expression in the localized tissue is the key regulator of the inflammatory process rather than its systemic blood levels.

The significantly lower expression levels of *TNFAIP3* in severe disease compared to mild disease is in accordance with a study by Jiang et al. [41], where the expression of *TNFAIP3* mRNA was reversely correlated with PASI score. Other studies showed an up-regulation of *TNFAIP3* expression in mild cases; this may be explained by the chronic inflammation existing in psoriasis patients [53, 54]. Some proinflammatory cytokines, such as TNF-α, IL-1, and IL-17, can stimulate peripheral blood mononuclear cells (PBMC), leading to activation of NF-κB which in turn enhances the transcription of the *TNFAIP3* gene [19, 55, 56]. Therefore, sustained inflammation accounts for the increased *TNFAIP3* expression in PBMC of patients with mild psoriasis. Such elevation has not been encountered in cases with severe psoriasis [41] although the proinflammatory cytokines are also increased in those patients [53].

The loss of up-regulation of *TNFAIP3* expression in severe psoriasis vulgaris can be explained by certain epigenetic events that may take place in the *TNFAIP3* gene including a 5′ region polymorphism or promoter methylation [26, 57]. Additionally, a haplotype TT>A polymorphic dinucleotide (T deletion followed by T to A transversion) linked to a decrease in *TNFAIP3* expression has been identified [27]. The other factor influencing *TNFAIP3* expression is promoter methylation. Worth mentioning, *TNFAIP3* is targeted by promoter methylation in various hematological malignancies. Accordingly, the inefficient expression of the *TNFAIP3* gene in severe psoriasis vulgaris may be due in part to its aberrant methylation [58, 59].

In this study, no correlations were detected between blood levels of *TNFAIP3* mRNA and each of: skin levels of *TNFAIP3* mRNA and skin levels of TNFAIP3 protein. These findings, together with the previously mentioned finding of absence of significant difference between blood *TNFAIP3* expression levels in mild and moderate to severe cases, confirm that *TNFAIP3* is expressed and acts specifically in tissues, irrespective of its blood levels, indicating that TNFAIP3 blood level is not a reliable indicator of disease severity in psoriatic patients. These findings also imply that reduced expression of *TNFAIP3* is a factor that contributes to the pathogenesis of psoriasis. However, it is not the only cause; external environmental stimuli are also required to precipitate a psoriatic reaction.

## Conclusions

The results of this study support previous human studies as well as experimental studies in mice which clearly suggest the association of *TNFAIP3* gene to psoriasis susceptibility and disease severity. Consequently, TNFAIP3 may serve as both a predictive and prognostic biomarker. Moreover, enhancing the expression and/or function of *TNFAIP3* gene provides a new line of treatment which needs to be confirmed by clinical trial studies. As the actions of TNFAIP3 become more apparent, its role in existent and future therapies for psoriasis becomes clearer. Further studies to understand the epigenetic events that affect TNFAIP3 regulation and the correlation between its levels and immunologic features of psoriasis are required.
